# Reactogenicity Correlates Only Weakly with Humoral Immunogenicity after COVID-19 Vaccination with BNT162b2 mRNA (Comirnaty^®^)

**DOI:** 10.3390/vaccines9101063

**Published:** 2021-09-24

**Authors:** Jürgen Held, Jan Esse, Koray Tascilar, Philipp Steininger, Kilian Schober, Pascal Irrgang, Rayya Alsalameh, Matthias Tenbusch, Christof Seggewies, Christian Bogdan

**Affiliations:** 1 Mikrobiologisches Institut-Klinische Mikrobiologie, Immunologie und Hygiene, Universitätsklinikum Erlangen und Friedrich-Alexander-Universität (FAU) Erlangen-Nürnberg, Wasserturmstr. 3/5, 91054 Erlangen, Germany; jan.esse@uk-erlangen.de (J.E.); kilian.schober@uk-erlangen.de (K.S.); rayya.alsalameh@uk-erlangen.de (R.A.); christian.bogdan@uk-erlangen.de (C.B.); 2 Medizinische Klinik 3-Rheumatologie und Immunologie & Deutsches Zentrum für Immuntherapie, Universitätsklinikum Erlangen und Friedrich-Alexander-Universität (FAU) Erlangen-Nürnberg, Ulmenweg 18, 91054 Erlangen, Germany; koray.tascilar@uk-erlangen.de; 3 Virologisches Institut-Klinische und Molekulare Virologie, Universitätsklinikum Erlangen und Friedrich-Alexander-Universität (FAU) Erlangen-Nürnberg, Schlossgarten 4, 91054 Erlangen, Germany; philipp.steininger@uk-erlangen.de (P.S.); pascal.irrgang@uk-erlangen.de (P.I.); matthias.tenbusch@uk-erlangen.de (M.T.); 4 Center for Medical Information and Communication Technology, Universitätsklinikum Erlangen, Krankenhausstraße 12, 91054 Erlangen, Germany; christof.seggewies@uk-erlangen.de

**Keywords:** SARS-CoV-2, mRNA vaccine, side effects, adverse reactions, adverse effects, antibody, machine learning, BioNTech, Pfizer

## Abstract

mRNA vaccines against severe acute respiratory syndrome coronavirus 2 (SARS-CoV-2), such as BNT162b2 (Comirnaty^®^), have proven to be highly immunogenic and efficient but also show marked reactogenicity, leading to adverse effects (AEs). Here, we analyzed whether the severity of AEs predicts the antibody response against the SARS-CoV-2 spike protein. Healthcare workers without prior SARS-CoV-2 infection, who received a prime-boost vaccination with BNT162b2, completed a standardized electronic questionnaire on the duration and severity of AEs. Serum specimens were collected two to four weeks after the boost vaccination and tested with the COVID-19 ELISA IgG (Vircell-IgG), the LIAISON^®^ SARS-CoV-2 S1/S2 IgG CLIA (DiaSorin-IgG) and the iFlash-2019-nCoV NAb surrogate neutralization assay (Yhlo-NAb). A penalized linear regression model fitted by machine learning was used to correlate AEs with antibody levels. Eighty subjects were enrolled in the study. Systemic, but not local, AEs occurred more frequently after the boost vaccination. Elevated SARS-CoV-2 IgG antibody levels were measured in 92.5% of subjects with Vircell-IgG and in all subjects with DiaSorin-IgG and Yhlo-NAb. Gender, age and BMI showed no association with the antibody levels or with the AEs. The linear regression model identified headache, malaise and nausea as AEs with the greatest variable importance for higher antibody levels (Vircell-IgG and DiaSorin-IgG). However, the model performance for predicting antibody levels from AEs was very low for Vircell-IgG (squared correlation coefficient r^2^ = 0.04) and DiaSorin-IgG (r^2^ = 0.06). AEs did not predict the surrogate neutralization (Yhlo-NAb) results. In conclusion, AEs correlate only weakly with the SARS-CoV-2 spike protein antibody levels after COVID-19 vaccination with BNT162b2 mRNA.

## 1. Introduction

Coronavirus disease 2019 (COVID-19) is caused by the severe acute respiratory syndrome coronavirus 2 (SARS-CoV-2), which was first described in December 2019 in Wuhan, China [1,2]. Since then, it has become a pandemic, infecting over 226 million people and resulting in approximately 4.6 million deaths worldwide [3]. Around one year after the pandemic outbreak, vaccination campaigns with the mRNA-based vaccines BNT162b2 (Comirnaty^®^, BioNTech/Pfizer, Mainz, Germany/New York City, NY, USA) and mRNA-1273 (Spikevax^®^, Moderna, Cambridge, MA, USA) have been carried out in several countries [4].

The authorization of new vaccines depends on their immunogenicity, clinical efficacy and safety [5]. The latter is usually assumed when the level of reactogenicity (i.e., transient signs of inflammation at the injection site (e.g., pain, redness, swelling and induration) or systemically (e.g., increase of body temperature, chills, fatigue, cephalgia or arthralgia)) is acceptable and severe AEs were not observed. In phase III randomized controlled trials, BNT162b2 has demonstrated an excellent clinical efficacy of 95.0% (95% CI: 90.3–97.6) in adults and 100% (95% CI: 75.3–100) in adolescents [6,7]. Accordingly, neutralizing antibodies, which are a surrogate for clinical efficacy, were three times higher than in convalescent individuals and were detected in approximately 97% of patients after the second injection of BNT162b2 [8,9,10,11].

In terms of safety, the frequency of mild-to-moderate adverse effects (AEs) of mRNA-based vaccines was substantial [6,12,13]. Immunized persons experienced a variety of local and systemic reactions following vaccination. In the post-marketing surveillance system of the Centers for Disease Control (CDC), 63.6% of vaccinees reported injection site pain, 29.1% fatigue, 24.7% headache, 17.0% myalgia and 7.0% fever after the first dose of BNT162b2. The side effects were even more pronounced after the second injection (66.5% injection site pain, 47.8% fatigue, 40.4% headache, 36.8% myalgia, 22.7% chills, 21.5% fever and 19.9% joint pain) [14]. Similar data on the efficacy, immunogenicity and reactogenicity were obtained with the mRNA-1273 vaccine [12,14,15,16].

Both medical doctors and vaccinees frequently ask the question, whether there is a correlation between vaccine reactogenicity and immunogenicity. Surprisingly, this question has only been poorly studied for any vaccine. Some published results point to a correlation of vaccine reactogenicity with the release of certain cytokines (e.g., tumor necrosis factor and macrophage migration inhibitory factor) but not with the antibody response [17,18,19]. 

In order to test for a correlation between reactogenicity and the antibody response against the SARS-CoV-2 spike protein, we initiated a prospective study amongst healthcare workers after a prime-boost vaccination with BNT162b2.

## 2. Materials and Methods

All employees of the Institute for Clinical Microbiology, Immunology and Hygiene of the University Hospital Erlangen, Germany who received a prime-boost vaccination with BNT162b2 in March and April 2021 and gave their informed consent were eligible for the study. Subjects were excluded if there was a history of previous SARS-CoV-2 infection or if the IgG antibodies targeting the SARS-CoV-2 nucleocapsid antigen (anti-SARS-CoV-2-NCP-ELISA IgG; Euroimmun AG, Lübeck, Germany), which were measured in all participants to rule out undetected SARS-CoV-2 infections, were positive.

Humoral immunogenicity data. The serum specimens were obtained two to four weeks after the boost vaccination. Anti-SARS-CoV-2 IgG antibodies targeting the spike antigen were measured with the COVID-19 ELISA IgG (Vircell-IgG; Vircell S.L., Granada, Spain) and the LIAISON^®^ SARS-CoV-2 S1/S2 IgG CLIA (DiaSorin-IgG; DiaSorin S.p.A., Saluggia, Italy). In addition, the iFlash-2019-nCoV NAb surrogate neutralization assay (Yhlo-NAb; Shenzhen YHLO Biotech Co., Ltd., Shenzhen, China) was used for quantification of the neutralizing antibody response. The Vircell-IgG uses a recombinant spike glycoprotein and nucleocapsid protein of SARS-CoV-2 as the coated antigens, whereas DiaSorin-IgG uses only the SARS-CoV-2 spike glycoprotein. Yhlo-NAb is based on the competition of the patient’s serum antibodies with recombinant angiotensin-converting-enzyme 2 for binding to the SARS-CoV-2 spike protein receptor-binding domain. The results of DiaSorin-IgG and Yhlo-NAb correlate closely with the conventional wild-type SARS-CoV-2 neutralization assays [20]. All assays were performed according to the manufacturers’ recommendations on automated platforms (Euroimmun Analyzer I, Vircell ThunderBolt, LIAISON XL and iFlash 1800 CLIA). The results were calculated as the antibody index (AI; Vircell-IgG) or expressed as arbitrary units/mL (AU/mL; DiaSorin-IgG and Yhlo-NAb). The Yhlo-NAb results above the upper validation limit were uniformly assigned a value of 10,000 AU/mL. The cut-off values were as follows: Vircell-IgG (<4 negative; 4–6 equivocal; >6 positive), DiaSorin-IgG (<12 negative; 12–15 equivocal; ≥15 positive) and Yhlo-NAb (<10 negative; ≥10 positive). 

Reactogenicity data. Adverse effects were extracted from a standardized electronic questionnaire that all study participants had to complete. The selection of typical vaccine-associated AEs, together with the temporal relationship to the vaccination itself, ensured that all analyzed AEs were directly related to the vaccine. The following data was collected: age, gender, height, body weight, known diseases, allergies, previous or subsequent COVID-19 infection, date of prime and boost vaccination, anti-inflammatory medications (e.g., ibuprofen) before and after the vaccinations, inability to work and occurrence of local and systemic vaccination AE with duration, severity and recovery. The following AE were listed in the questionnaire: pain, swelling, redness, pruritus, rash and urticaria at the injection site, local lymphadenopathy, chills, malaise, nausea, vomiting, fatigue, headache, pain in the limbs, joint pain, muscle pain, elevated body temperature in °C, anaphylaxis, allergies, facial paresis and other discomforts. The severity was assessed using a numerical rating scale from 0 to 10, in which 0 stands for no AE and 10 for the worst imaginable intensity of the AE.

The study was approved by the Ethics Committee of the Friedrich Alexander University of Erlangen-Nürnberg (277_21 Bc). Written informed consent was obtained from all the participants. 

The statistical analysis was performed using SPSS-V24 (IBM Corp., Armonk, NY, USA) and R-V4 (R Foundation for Statistical Computing, Vienna, Austria). The means were given with the standard deviation (SD) and medians with an interquartile range (IQR). AEs that occurred in less than five subjects were excluded from the analysis. For comparison of the mean antibody levels in subjects with and without AE, the Mann–Whitney *U* test was used. Differences with *p*-values < 0.05 were considered significant. To include both the severity and duration of an AE into the analysis, we calculated the area under the severity–time curve (AUC), i.e., the product of the severity and duration of an AE. The total AE burden (AUC_total_) was equal to the sum of all AUCs of a participant. We used Pearson’s correlation coefficient to describe the crude association between the AUC or the AUC_total_ and the antibody levels. Subsequently, we aimed to identify the relative power of influence of individual AEs in the overall association. Since conventional analyses of association based on least squares methods (i.e., correlation) are likely to overfit the data, we applied a method that could also address this problem as a sensitivity analysis. Therefore, we used penalized linear regression with nested cross-validation to predict the antibody results from the AUC values of individual AEs. This analysis was done only on the Vircell- and DiaSorin-IgG results, because the Yhlo-NAb results did not significantly correlate with the AUC_total_. For model training and evaluation, a nested cross-validation was performed in four cycles. In each of these four cycles, three randomly selected parts of the data were used to generate five resampled (bootstrapped) datasets, and we fitted a number of models to find the optimal hyperparameters using a grid search method for the regularization (alpha) and penalty (lambda) hyperparameters. After the model was tuned in this way, we estimated a variable importance of the training data and averaged the variable importance from the four cross-validation cycles. Finally, the model performance was assessed by making predictions on the remaining one-fourth of the data not used for model training, and we averaged the performance metrics, i.e., root mean squared error (RMSE) and r^2^, from each cross-validation cycle to estimate the overall performance.

## 3. Results

Eighty subjects (25 male, 55 female, mean age 44.7 +/− 13.7 years and mean body mass index (BMI) 25.1 +/− 4.3 kg/m^2^) were included in the study. The mean interval between the prime and boost vaccinations was 23 +/− 2 days and between the boost vaccination and blood sampling was 15.9 +/− 3.9 days. None of the study participants reported to suffer from immunocompromising diseases or to take immunosuppressive medications able to influence antibody production.

There were no significant differences in the frequency of the local AEs at the injection site after the prime and boost vaccinations. However, axillary lymphadenopathy and seven out of nine systemic AEs were reported significantly more often after the boost vaccination. The reactogenicity data are summarized in Table 1.

**Figure 1:**
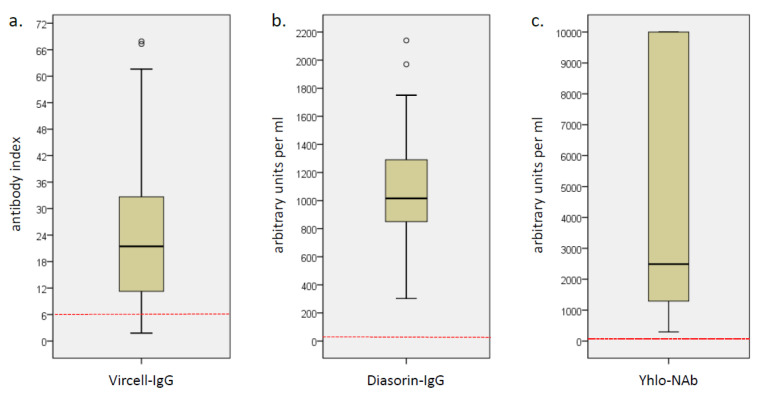
Box plots of the SARS-CoV-2 antibody levels measured with (**a**) Vircell-IgG, (**b**) DiaSorin-IgG and (**c**) Yhlo-NAb. The red dotted line shows the threshold for positivity. Open circles depict outliers.

All subjects had positive SARS-CoV-2 antibody levels in DiaSorin-IgG (median 1011 AU/mL, IQR 860–1290) and Yhlo-NAb (median 2489 AU/mL, IQR 1293–10,000). In Vircell-IgG, 74 subjects (92.5%) had positive, 3 equivocal and 3 negative antibody levels (median 21.5 AI, IQR 11.3–32.7) (Figure 1). The results of Vircell-IgG and DiaSorin-IgG showed a strong correlation (r = 0.501, *p* < 0.001), whereas there was no significant correlation between Vircell-IgG and Yhlo-NAb and only a correlation with a small effect size between DiaSorin-IgG and Yhlo-NAb (r = 0.364, *p* < 0.001; Figure S1).

Gender, age and BMI did not show an association with the antibody levels or with the AUCs of the AEs (Table S1). An exception was muscle pain after the boost vaccination that correlated significantly with age (r = 0.235, *p* = 0.036). In contrast, subjects who took anti-inflammatory medication after the boost dose had significantly higher AUCs of injection site pain, chills, malaise, fatigue, headache, pain in the limbs and elevated temperature than patients who did not. The antibody levels were not affected by this use of anti-inflammatory drugs (Table S2). 

A comparison of the mean antibody levels in subjects with and without a specific AE revealed the following (Table 2): After the prime vaccination, only subjects with headaches had (significantly) higher antibody levels than those without (Vircell-IgG: 26.7 vs. 17.9 AI, *p* = 0.042; DiaSorin IgG: 1310 vs. 1005 AU/mL, *p* = 0.011; Yhlo-NAb: 6689 vs. 2345 AU/mL, *p* = 0.062). After the boost vaccination, the mean Vircell-IgG levels were significantly higher in subjects suffering from malaise (29.1 vs. 18.6 AI, *p* = 0.027) and fatigue (22.4 vs. 15.1 AI, *p* = 0.039), whereas the DiaSorin-IgG levels were significantly higher in subjects with local lymphadenopathy (1220 vs. 1000 AU/mL, *p* = 0.041) and nausea (1310 vs. 1010 AU/mL, *p* = 0.036). Importantly, however, the Yhlo-NAb surrogate neutralization assay did not reveal significantly elevated antibody levels in any of the patient groups.

**Figure 2:**
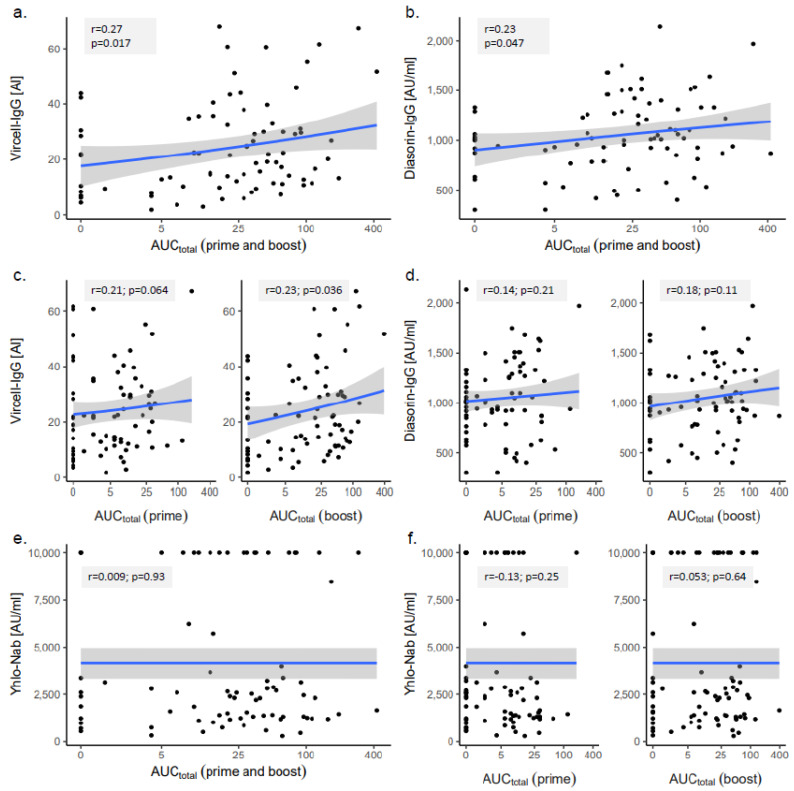
Correlation analysis of the total adverse effect burden (AUC_total_) and antibody levels measured with (**a**,**c**) Vircell-IgG, (**b**,**d**) DiaSorin-IgG and (**e**,**f**) Yhlo-NAb. r, Pearson’s correlation coefficient.

A correlation analysis between the total AE burden (AUC_total_) and the antibody levels showed a significant correlation with a small effect size for the Vircell-IgG (r = 0.27, *p* = 0.017) and the DiaSorin-IgG results (r = 0.23, *p* = 0.047). Considering the timing of the AEs (prime versus boost), only the AUC_total_ after the boost vaccination correlated significantly with the Vircell-IgG results (r = 0.23, *p* = 0.036, Figure 2).

**Figure 3:**
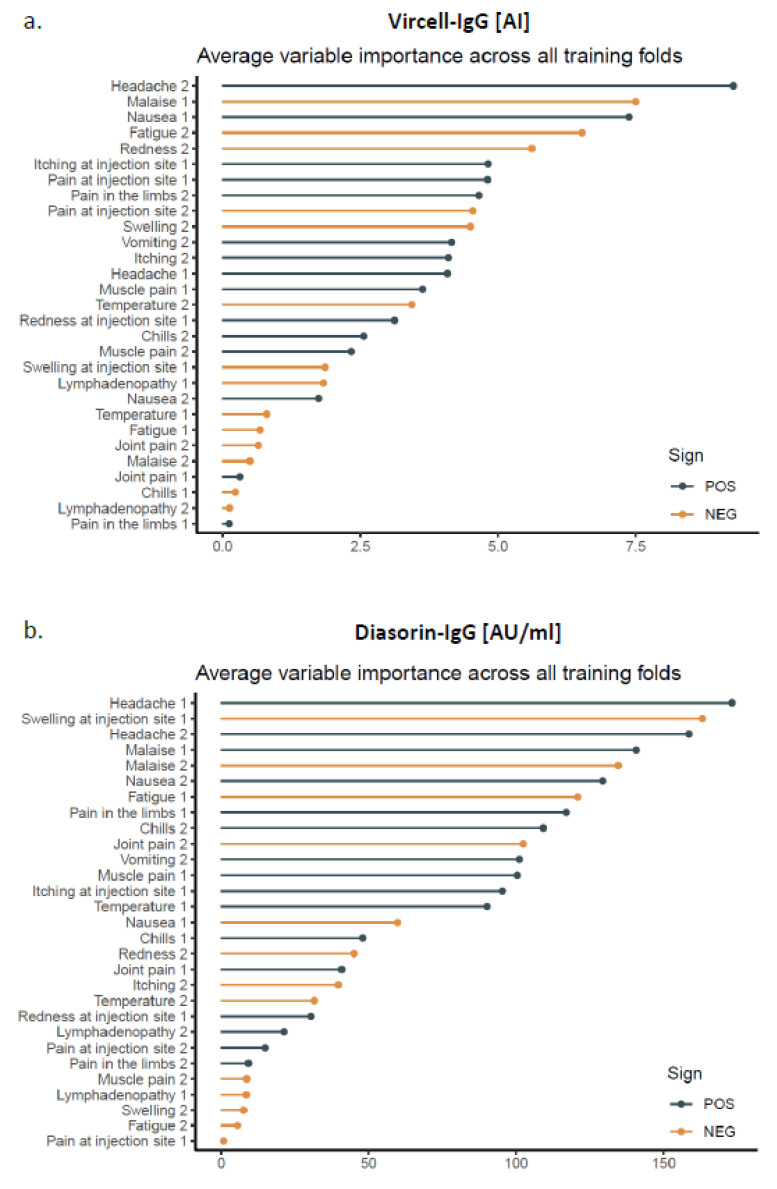
Average variable importance for the prediction of antibody levels measured with (**a**) Vircell-IgG and (**b**) DiaSorin-IgG from the adverse effects. Lines in grey or lines in orange represent adverse effects that show a positive or negative correlation with the antibody levels, respectively. The number behind the adverse effects refers to the time point of the occurrence (1 = after prime vaccination and 2 = after boost vaccination).

In order to examine the influence of the various AEs on the antibody levels, we used a penalized linear regression model fitted by machine learning. The AEs with the highest variable importance that positively influenced the SARS-CoV-2 antibody levels were headache, malaise and nausea (Figure 3). However, the model performance to predict the antibody levels from the AUCs was very low for both Vircell-IgG (RMSE 32.3 +/− 8.0, r^2^ = 0.04 +/− 0.02) and DiaSorin-IgG (RMSE 1892 +/− 1258, r^2^ = 0.06 +/− 0.06).

## 4. Discussion

In the present study, we collected the reactogenicity and immunogenicity data of 80 subjects after a prime-boost vaccination with BNT162b2 mRNA in order to analyze whether the magnitude of the AEs predicted the extent of the SARS-CoV-2 antibody response.

Our data showed that almost all subjects had measurable SARS-CoV-2 antibody levels two weeks after receiving the second vaccine dose and that systemic AEs were much more common after the boost vaccination. This is in line with previously published data [4,6,12,14].

While gender, age and BMI affected neither the antibody levels nor the magnitude of the AEs, the occurrence of various AEs correlated with the use of anti-inflammatory medications after the boost vaccination. This finding most likely results from the intake of anti-inflammatory drugs by subjects with severe AEs rather than from drug-induced side effects. At the same time, this observation argues for the validity of our overall adverse effect assessment by summarizing the severity and duration of the adverse effects in a single metric. Another important finding is that the use of anti-inflammatory drugs after a vaccination did not affect the antibody levels.

The correlation analysis between the total AE burden and SARS-CoV-2 antibody levels showed a significant correlation with a small effect size only for the Vircell-IgG and DiaSorin-IgG results. In contrast, neutralizing the antibody levels, as measured by the Yhlo-NAb surrogate neutralization assay, did not show such a correlation, which seems plausible, because they reflected only part of the antibody response. As the Yhlo-NAb results above the upper validation limit of the assay were uniformly assigned a value of 10.000 AU/mL, we formally cannot exclude that a recalculation with the exact values for these very high Yhlo-NAb results might reveal some degree of correlation between the AEs and neutralizing antibodies. However, the total AE burden of these very high Yhlo-NAb results ranges between low to middle values after the prime vaccination and is evenly distributed between low and high values after the boost vaccination, which makes a correlation between the AEs and neutralizing antibodies very unlikely (Figure 2e,f).

The comparison of the mean antibody levels in patients with and without certain AEs demonstrated that the antibody levels were significantly higher in subjects suffering from headache, malaise, fatigue, local lymphadenopathy and nausea. However, these differences were not consistent for the prime and boost vaccinations or for different antibody assays. Therefore, it is unlikely that the appearance of these AEs has a profound influence on the antibody response. This hypothesis is supported by the results of the penalized linear regression model, which revealed that headache, malaise and nausea were the AEs with the highest variable importance, but the overall performance in predicting the antibody levels from the AUCs was very low. Only 4% and 6% of the variations of the Vircell-IgG and DiaSorin-IgG antibody levels, respectively, were explained by variations in the AEs. 

A similar conclusion was recently reached by Hwang et al., who also studied the relationship between the reactogenicity and immunogenicity after the COVID-19 vaccination but used only one commercially available ELISA to determine the SARS-CoV-2 IgG response against the S1 domain of the spike protein [19]. Their cohort of vaccinees consisted of 135 healthy adults, who had either received one dose of AZD1222 (Vaxcevria^®^, AstraZeneca/Oxford, *n* = 42) or two doses of BNT162b2 (*n* = 93). The degree of AEs was not significantly associated with the S1-SARS-CoV-2 IgG levels in the AZD1222 or the BNT162b2 groups.

An efficient immune response to a vaccine requires intimate interactions between the components of the innate and adaptive immune system [21]. Vaccines, which contain microbial adjuvants (e.g., monophosphoryl-lipid A) that strongly stimulate pattern recognition molecules of innate immune cells, such as dendritic cells and macrophages, are not only highly immunogenic but also exhibit a rather strong reactogenicity (e.g., Cervarix^®^, Fendrix^®^ or Shingrix^®^) [22,23,24,25]. These and other observations have supported the “no pain, no gain” concept [26]. mRNA vaccines formulated in lipid nanoparticles (LNP) exhibit a strong endogenous adjuvant activity, which is due to the LNPs, the mRNA and nucleic acid byproducts from mRNA synthesis [27,28]. On the other hand, the prophylactic intake of anti-inflammatory drugs such as paracetamol or cyclooxygenase inhibitors had only a limited inhibitory effect on the antibody response to various adjuvant-containing vaccines, including diphtheria, tetanus, pneumococcal or hepatitis B vaccines, which was much weaker or even gone after the second vaccine dose [29]. Thus, a reactogenic, inflammatory innate immune response is not a prerequisite for obtaining an adequate adaptive immune response. This notion is strongly supported by our present findings that AEs correlated only weakly with the SARS-CoV-2 antibody levels after a COVID-19 vaccination with BNT162b2 mRNA and that the intake of anti-inflammatory drugs did not affect the anti-SARS-CoV-2 antibody response. Current and future systems’ immunology approaches aim to dissect the signals that account for AEs and that are required for immunogenicity and clinical vaccine efficacy [29,30].

## 5. Conclusions

Elevated SARS-CoV-2 IgG antibody levels were measured in nearly all subjects after the COVID-19 vaccination with BNT162b2 mRNA (Comirnaty^®^). AEs were frequent and systemic but not local, AEs occurred more often after the boost vaccination. There was only a weak correlation between the AEs and SARS-CoV-2 antibody levels. Consequently, it was not possible to predict the antibody levels from the frequency and/or severity of the AEs.

## Figures and Tables

**Table 1:** Frequency of the adverse effects after the prime and boost vaccinations.

Type of Adverse Effect	After Prime Dose (Yes/No) (% Yes)	After Boost Dose (Yes/No) (% Yes)	*p*-Value
any adverse effect	59/21 (73.8)	66/14 (82.5)	0.143
inability to work	6/74 (7.5)	21/59 (26.3)	**<0.001**
any local injection site reaction	54/26 (67.5)	59/21 (73.8)	0.302
pain	52/28 (65.0)	57/23 (71.3)	0.359
swelling	7/73 (8.8)	11/69 (13.8)	0.344
redness	5/75 (6.3)	5/75 (6.3)	1.000
itching	2/78 (2.5)	2/78 (2.5)	1.000
local lymphadenopathy	2/78 (2.5)	12/68 (15.0)	**0.006**
any systemic reaction	28/52 (35.0)	51/29 (63.7)	**<0.001**
chills	1/79 (1.3)	15/65 (18.8)	**0.001**
malaise	5/75 (6.3)	22/58 (27.5)	**<0.001**
nausea	4/76 (5.0)	6/74 (7.5)	0.687
fatigue	17/63 (21.3)	43/37 (53.8)	**<0.001**
headache	10/70 (12.5)	29/51 (36.3)	**<0.001**
pain in the limbs	3/77 (3.8)	22/58 (27.5)	**<0.001**
joint pain	2/78 (2.5)	5/75 (6.3)	0.375
muscle pain	2/78 (2.5)	15/65 (18.8)	**0.001**
elevated body temperature	1/79 (1.3)	9/71 (11.3)	**0.021**

Systemic, but not local, adverse effects are significantly more common after the boost dose compared to the prime dose. Significant results are highlighted in bold.

**Table 2:** Median SARS-CoV-2 antibody levels stratified after the occurrence of adverse effects.

Type of Adverse Effect	SARS-CoV-2 Antibody Assay	Subjects with Adverse Events after Prime Dose		*p*-Value	Subjects with Adverse Events after Boost Dose		*p*-Value
		yes	no		yes	no	
any adverse effect	Vircell-IgG (AI)	22.3	17.2	0.167	21.5	21.4	0.147
	DiaSorin-IgG (AU/mL)	1060	1000	0.116	1020	1011	0.625
	YHLO-Nab (AU/mL)	2451	2526	0.519	2555	1991	0.421
inability to work	Vircell-IgG (AI)	15.2	21.7	0.448	23.5	21.5	0.158
	DiaSorin-IgG (AU/mL)	1016	986	0.619	1060	1011	0.660
	YHLO-Nab (AU/mL)	1245	2587	0.051	2305	2583	0.856
any local injection site reactions	Vircell-IgG (AI)	21.9	19.0	0.670	19.6	22.2	0.611
	DiaSorin-IgG (AU/mL)	1035	1010	0.439	1015	1021	0.955
	YHLO-Nab (AU/mL)	2350	2592	0.709	2583	1866	0.450
pain	Vircell-IgG (AI)	20.8	20.3	0.813	19.6	22.2	0.754
	DiaSorin-IgG (AU/mL)	1020	1011	0.803	1015	1021	0.913
	YHLO-Nab (AU/mL)	2423	2558	0.951	2590	1601	0.171
swelling	Vircell-IgG (AI)	22.4	20.1	0.319	20.1	21.9	0.955
	DiaSorin-IgG (AU/mL)	1060	1011	0.675	1000	1020	0.796
	YHLO-Nab (AU/mL)	2874	2451	0.925	3377	2381	0.371
redness	Vircell-IgG (AI)	11.4	21.4	0.985	11.4	21.9	0.196
	DiaSorin-IgG (AU/mL)	921	1020	0.937	903	1020	0.370
	YHLO-Nab (AU/mL)	2793	2451	0.992	2793	2395	0.637
local lymphadenopathy	Vircell-IgG (AI)	---	---	---	29.1	18.9	0.163
	DiaSorin-IgG (AU/mL)	---	---	---	1220	1000	**0.041**
	YHLO-Nab (AU/mL)	---	---	---	2795	2461	0.659
any systemic reactions	Vircell-IgG (AI)	26.7	16.1	**0.037**	22.4	13.5	**0.015**
	DiaSorin-IgG (AU/mL)	1255	958	**0.014**	1060	943	0.109
	YHLO-Nab (AU/mL)	2730	2345	0.189	2583	2381	0.189
chills	Vircell-IgG (AI)	---	---	---	28.2	20.3	0.147
	DiaSorin-IgG (AU/mL)	---	---	---	1185	1000	0.050
	YHLO-Nab (AU/mL)	---	---	---	2702	2395	0.471
malaise	Vircell-IgG (AI)	29.1	20.1	0.311	29.1	18.6	**0.027**
	DiaSorin-IgG (AU/mL)	1330	1011	0.370	1100	1010	0.318
	YHLO-Nab (AU/mL)	1460	2526	0.976	2416	2517	0.983
nausea	Vircell-IgG (AI)	---	---	---	29.9	20.1	0.289
	DiaSorin-IgG (AU/mL)	---	---	---	1310	1010	**0.036**
	YHLO-Nab (AU/mL)	---	---	---	2558	2489	0.593
fatigue	Vircell-IgG (AI)	28.4	18.9	0.171	22.4	15.1	**0.039**
	DiaSorin-IgG (AU/mL)	1250	1010	0.490	1050	958	0.257
	YHLO-Nab (AU/mL)	1601	2526	0.863	2526	2395	0.599
headache	Vircell-IgG (AI)	26.9	17.9	**0.042**	22.2	21.4	0.152
	DiaSorin-IgG (AU/mL)	1310	1005	**0.011**	1020	1010	0.339
	YHLO-Nab (AU/mL)	6689	2345	0.062	2650	2381	0.443
pain in the limbs	Vircell-IgG (AI)	---	---	---	22.5	21.4	0.217
	DiaSorin-IgG (AU/mL)	---	---	---	1110	1000	0.395
	YHLO-Nab (AU/mL)	---	---	---	2788	2343	0.370
joint pain	Vircell-IgG (AI)	---	---	---	20.1	21.5	0.847
	DiaSorin-IgG (AU/mL)	---	---	---	869	1020	0.158
	YHLO-Nab (AU/mL)	---	---	---	1660	2526	0.652
muscle pain	Vircell-IgG (AI)	---	---	---	22.3	21.4	0.284
	DiaSorin-IgG (AU/mL)	---	---	---	1010	1020	0.965
	YHLO-Nab (AU/mL)	---	---	---	2810	2451	0.926
elevated temperature	Vircell-IgG (AI)	---	---	---	26.8	21.4	0.548
	DiaSorin-IgG (AU/mL)	---	---	---	1050	1011	0.713
	YHLO-Nab (AU/mL)	---	---	---	2197	2526	0.461

Subjects with certain adverse effects have significantly higher mean antibody levels compared to subjects without these adverse effects using the Vircell-IgG and/or the DiaSorin-IgG assay but not with the Yhlo-NAb surrogate neutralization assay. Adverse effects that occurred in less than five subjects were excluded from the analysis. Significant results are highlighted in bold. AI, antibody index; AU, arbitrary units.

## Data Availability

The original datasets of this study are available from the corresponding author (J.H.) upon reasonable request.

## Supplementary Materials

The following are available online at https://www.mdpi.com/article/10.3390/vaccines9101063/s1: Figure S1: Scatter plots of the SARS-CoV-2 antibody results measured by three different assays. Table S1: (**a**) Comparison of the mean antibody levels of woman and men, (**b**) median AUCs of the adverse effects stratified by gender, (**c**) correlation of the antibody levels with age and body mass index and (**d**) correlation of the adverse effects with age and body mass index. Table S2: (**a**) Median AUCs stratified after taking anti-inflammatory medication, and (**b**) median SARS-CoV-2 antibody levels stratified after taking anti-inflammatory medication.
