# Supplementary material for: Reactogenicity Correlates Only Weakly with Humoral Immunogenicity after COVID-19 Vaccination with BNT162b2 mRNA (Comirnaty®)
Source: Vaccines (Basel). 2021 Sep 24;9(10):1063. doi: 10.3390/vaccines9101063 (PMC8539109; doi:10.3390/vaccines9101063)
Supplement: Supplementary file 1 [file vaccines-09-01063-s001.zip › vaccines-1377139-supplementary.pdf]

Held *et al*. Reactogenicity correlates only weakly with humoral immunogenicity after COVID-19 vaccination with BNT162b2 mRNA.

Supplementary materials

Figure S1

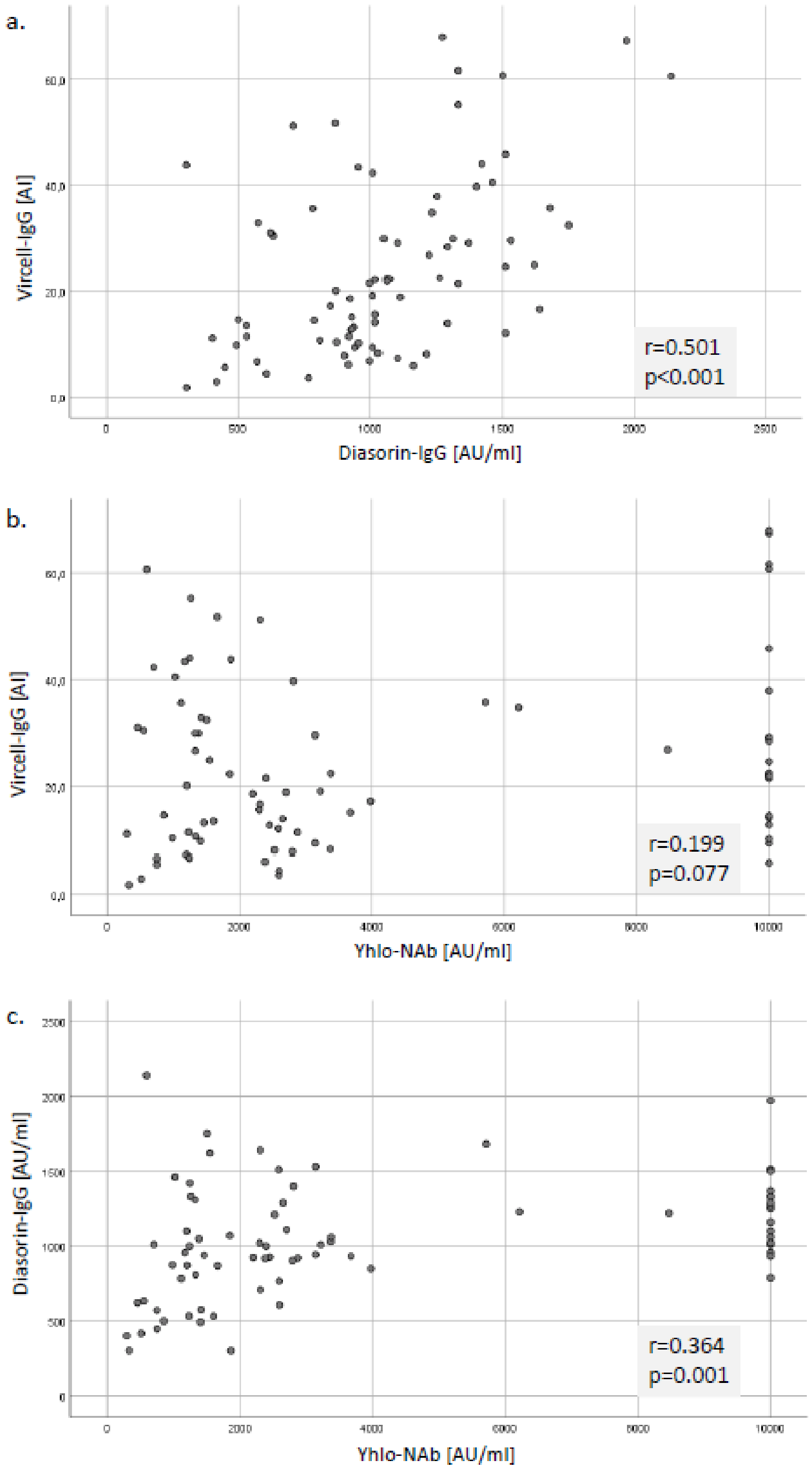

Figure legend: Scatter plots of SARS-CoV-2 antibody results measured by three different assays. Pearson's correlation coefficient r is stated in the grey box.

Table S1a: Comparison of mean antibody levels of woman and men

	women	men	p-value
**Vircell-IgG [AI] (IQR)**	21.7 (11.4-35.6)	15.1 (9.2-28.0)	0.229
**Diasorin-IgG [AU/ml] (IQR)**	1025 (871-1310)	949 (778-1250)	0.378
**Yhlo-Nab [AU/ml] (IQR)**	2517 (1324-10000)	2196 (1250-3824)	0.473

Table legend: Median SARS-CoV-2 antibody levels of woman and men and comparison of means with the Mann-Whitney-U-test. AI, antibody index; AU, arbitrary units; IQR, interquartile range.

Table S1b: Median AUCs of adverse effects stratified after gender

		median AUC after prime dose (IQR)	p-value	median AUC after boost dose (IQR)	p-value
**inability to work [d]**	women	0 (0-0)	0.701	0 (0-1)	0.163
	men	0 (0-0)		0 (0-0)	
**local injection site reactions**					
**pain**	women	2 (0-8.5)	0.444	3 (0-11)	0.768
	men	4 (0-6.0)		4 (2-9)	
**swelling**	women	0 (0-0)	0.213	0 (0-0)	0.323
	men	0 (0-0)		0 (0-0)	
**redness**	women	0 (0-0)	0.656	0 (0-0)	0.433
	men	0 (0-0)		0 (0-0)	
**local lymphadenopathy**	women	---	---	0 (0-0)	0.531
	men	---	---	0 (0-0)	
**systemic reactions**					
**chills**	women	---	---	0 (0-0)	0.240
	men	---	---	0 (0-0)	
**malaise**	women	0 (0-0)	0.148	0 (0-4)	0.888
	men	0 (0-0)		0 (0-0)	
**nausea**	women	---	---	0 (0-0)	0.433
	men	---	---	0 (0-0)	
**fatigue**	women	0 (0-0)	0.709	5 (0-14)	0.053
	men	0 (0-0)		0 (0-5)	
**headache**	women	0 (0-0)	0.421	0 (0-5.5)	0.731
	men	0 (0-0)		0 (0-3)	
**pain in the limbs**	women	---	---	0 (0-4)	0.317
	men	---	---	0 (0-0)	
**joint pain**	women	---	---	0 (0-0)	0.647
	men	---	---	0 (0-0)	
**muscle pain**	women	---	---	0 (0-0)	0.636
	men	---	---	0 (0-0)	
**elevated temperature**	women	---	---	0 (0-0)	0.228
	men	---	---	0 (0-0)	

Table legend: Median area under the adverse effect curve (AUC) for each adverse effect after prime and boost vaccination stratified after gender. Adverse effects (AEs) that occurred in less than five subjects were excluded from the analysis. IQR, interquartile range.

Table S1c: Correlation of antibody levels with age and body mass index

		Pearson's correlation coefficient r	p-value
**Vircell-IgG**	age	0.062	0.584
	BMI	0.081	0.480
**Diasorin-IgG**	age	-0.153	0.182
	BMI	0.077	0.510
**Yhlo-Nab**	age	-0.066	0.562
	BMI	0.162	0.156

Table S1d: Correlation of adverse effects with age and body mass index

		Pearson's correlation coefficient r after prime dose	p-value	Pearson's correlation coefficient r after boost dose	p-value
**inability to work [d]**	age	-0.053	0.643	-0.052	0.645
	BMI	-0.014	0.904	-0.072	0.529
**local injection site reactions**					
**pain**	age	-0.003	0.978	0.000	0.996
	BMI	-0.016	0.889	-0.051	0.660
**swelling**	age	0.040	0.724	0.009	0.938
	BMI	0.084	0.464	0.190	0.095
**redness**	age	0.032	0.778	0.054	0.637
	BMI	0.102	0.374	0.132	0.251
**local lymphadenopathy**	age	---	---	-0.160	0.155
	BMI	---	---	-0.041	0.719
**systemic reactions**					
**chills**	age	---	---	0.066	0.559
	BMI	---	---	-0.061	0.596
**malaise**	age	-0.058	0.612	0.023	0.841
	BMI	0.101	0.381	-0.089	0.439
**nausea**	age	---	---	0.102	0.367
	BMI	---	---	-0.008	0.943
**fatigue**	age	-0.144	0.203	0.083	0.466
	BMI	0.037	0.750	-0.070	0.543
**headache**	age	-0.040	0.722	-0.006	0.956
	BMI	0.182	0.110	0.005	0.966
**pain in the limbs**	age	---	---	0.031	0.783
	BMI	---	---	-0.008	0.945
**joint pain**	age	---	---	0.075	0.508
	BMI	---	---	-0.066	0.565
**muscle pain**	age	---	---	0.235	0.036
	BMI	---	---	-0.066	0.567
**elevated temperature**	age	---	---	0.006	0.954
	BMI	---	---	-0.193	0.090

Table legend: Adverse effects (AEs) that occurred in less than five subjects were excluded from the analysis. Significant correlations are marked in orange. BMI, body mass index; r, Pearson's correlation coefficient.

Table S2a: Median AUCs stratified after taking anti-inflammatory medication

	anti-inflammatory med. after prime dose		p-value	anti-inflammatory med. after boost dose		p-value
	yes (n=2)	no (n=78)		yes (n=22)	no (n=58)	
**inability to work [d]**	---	0	---	1	0	<0.001
**local injection site reaction after prime and boost dose, respectively [AUC]**						
**pain**	25.5	2	0.016	12	2	<0.001
**swelling**	6	0	0.306	0	0	0.145
**redness**	6	0	0.292	0	0	0.258
**local lymphadenopathy [AUC]**	---	---	---	0	0	0.202
**systemic reactions [AUC]**						
**chills**	---	---	---	2	0	<0.001
**malaise**	10	0	0.306	4	0	<0.001
**nausea**	---	---	---	0	0	0.207
**fatigue**	25	0	0.006	10	0	<0.001
**headache**	16	0	0.320	7	0	<0.001
**pain in the limbs**	---	---	---	2	0	0.003
**joint pain**	---	---	---	0	0	0.090
**muscle pain**	---	---	---	0	0	0.070
**elevated temperature**	---	---	---	0	0	<0.001

Table legend: Median area under the adverse effect curve (AUC) for each adverse effect after prime and boost vaccination stratified after use of anti-inflammatory medication. Adverse effects (AEs) that occurred in less than five subjects were excluded from the analysis. Significant correlations are marked in orange.

Table S2b: Median SARS-CoV-2 antibody levels stratified after taking anti-inflammatory medication

	anti-inflammatory med. after prime dose		p-value	anti-inflammatory med. after boost dose		p-value
	yes (n=2)	no (n=78)		yes (n=22)	no (n=58)	
**Vircell-IgG [AI] (IQR)**	49.2 (31.0-67.3)	19.6 (10.9-32.7)	0.084	20. (14.6-31.0)	21.4 (10.2-34.8)	0.314
**Diasorin-IgG [AU/ml] (IQR)**	1297 (623-1970)	1016 (860-1290)	0.747	1020 (869-1330)	1011 (810-1270)	0.757
**Yhlo-NAb [AU/ml] (IQR)**	5229 (458-10000)	2489 (1293-9234)	0.820	2526 (1414-3976)	2395 (1237-10000)	0.583

Table legend: Median SARS-CoV-2 antibody levels stratified after taking anti-inflammatory medication and comparison of means with the Mann-Whitney-U-test. AI, antibody index; AU, arbitrary units; IQR, interquartile range.
